# Long-term follow up of patients with WHO grade 2 oligodendroglioma

**DOI:** 10.1007/s11060-023-04368-6

**Published:** 2023-08-21

**Authors:** Louise Carstam, Francesco Latini, Ole Solheim, Jiri Bartek, Lars K. Pedersen, Maria Zetterling, Stanislav Beniaminov, Kristin Sjåvik, Mats Ryttlefors, Margret Jensdottir, Bertil Rydenhag, Anja Smits, Asgeir S. Jakola

**Affiliations:** 1 Department of Neurosurgery, Sahlgrenska University Hospital, Blå Stråket 5, 41345 Göteborg, Sweden; 2 Institution of Neuroscience and Physiology, Sahlgrenska Academy, Gothenburg University, Göteborg, Sweden; 3 Department of Medical Sciences, Section of Neurosurgery, Uppsala University Hospital, Uppsala, Sweden; 4 Department of Neurosurgery, St. Olavs University Hospital, Trondheim, Norway; 5 Department of Neuromedicine and Movement Science, Norwegian University of Science and Technology, Trondheim, Norway; 6 Department of Clinical Neuroscience, Section for Neurosurgery, Karolinska Institutet and Department of Neurosurgery, Karolinska University Hospital, Stockholm, Sweden; 7 Department of Neurosurgery, Rigshospitalet, Copenhagen, Denmark; 8 Department of Neurosurgery, University Hospital of North Norway, Tromsø, Norway; 9 Department of Neurology, Karolinska University Hospital, Stockholm, Sweden; 10 Department of Neurology, Sahlgrenska University Hospital, Göteborg, Sweden

**Keywords:** Oligodendrogliomas, *IDH*-mutation, 1p19q-codeletion, Survival, Low-grade gliomas

## Abstract

**Purpose:**

Since the introduction of the molecular definition of oligodendrogliomas based on isocitrate dehydrogenase (*IDH*)-status and the 1p19q-codeletion, it has become increasingly evident how this glioma entity differs much from other diffuse lower grade gliomas and stands out with longer survival and often better responsiveness to adjuvant therapy. Therefore, apart from using a molecular oligodendroglioma definition, an extended follow-up time is necessary to understand the nature of this slow growing, yet malignant condition. The aim of this study was to describe the long-term course of the oligodendroglioma disease in a population-based setting and to determine which factors affect outcome in terms of survival.

**Methods:**

All adults with WHO-grade 2 oligodendrogliomas with known 1p19q-codeletion from five Scandinavian neurosurgical centers and with a follow-up time exceeding 5 years, were analyzed regarding survival and factors potentially affecting survival.

**Results:**

126 patients diagnosed between 1998 and 2016 were identified. The median follow-up was 12.0 years, and the median survival was 17.8 years (95% CI 16.0–19.6).

Factors associated with shorter survival in multivariable analysis were age (HR 1.05 per year; CI 1.02–1.08, *p* < 0.001), tumor diameter (HR 1.05 per millimeter; CI 1.02–1.08, *p* < 0.001) and poor preoperative functional status (KPS < 80) (HR 4.47; CI 1.70–11.78, *p* = 0.002). In our material, surgical strategy was not associated with survival.

**Conclusion:**

Individuals with molecularly defined oligodendrogliomas demonstrate long survival, also in a population-based setting. This is important to consider for optimal timing of therapies that may cause long-term side effects. Advanced age, large tumors and poor function before surgery are predictors of shorter survival.

**Graphical Abstract:**

**Supplementary Information:**

The online version contains supplementary material available at 10.1007/s11060-023-04368-6.

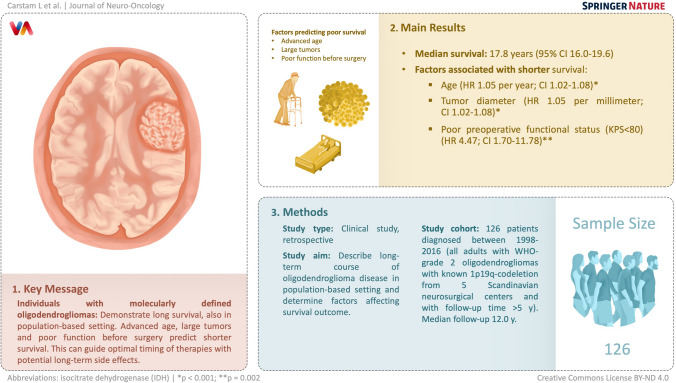

## Introduction

Oligodendrogliomas are usually slow-growing primary CNS tumors that often give rise to first-time seizures in young to middle-aged adults. The tumors are classified as diffuse lower grade gliomas (LGG) together with isocitrate dehydrogenase (*IDH)*-mutated astrocytomas [1]. The tradition for treating oligodendrogliomas and astrocytomas together in the scientific literature is, however, likely to have blurred important differences between the respective subgroups.

The advent of molecular definitions in tumor classification has allowed clear demarcations between subtypes and elucidated important differences in anatomical preferential locations, clinical course, treatment responses and prognosis. For oligodendrogliomas, predictors for an unfavorable clinical course are particularly at risk for being concealed in merged analyses due to dominant effects from tumor subtypes with shorter time to event, such as astrocytomas and *IDH*-wildtype (*IDH*-wt) LGG, all being part of older LGG cohorts [2]. Another shortcoming, common to most publications with molecular data, is that the follow-up time of patients with oligodendrogliomas is too short to adequately assess survival [2, 3]. Surrogate markers for survival such as "progression free survival" (PFS) have been used to circumvent this problem, but the correlation between PFS and actual survival may be very weak [4–8]. Since oligodendrogliomas are rare tumors, large cohorts with detailed individual level data are needed but still few [9]. Other studies with relative long follow-up may reflect the clinical course for patients selected for surgery in specialized centers [7, 10].

To address these difficulties, we performed a long-term multicenter study including only patients with molecularly defined grade 2 oligodendrogliomas, with the aim to describe the course of the disease and to determine prognostics in a population-based context.

## Materials and methods

### Study population

**Fig. 1:**
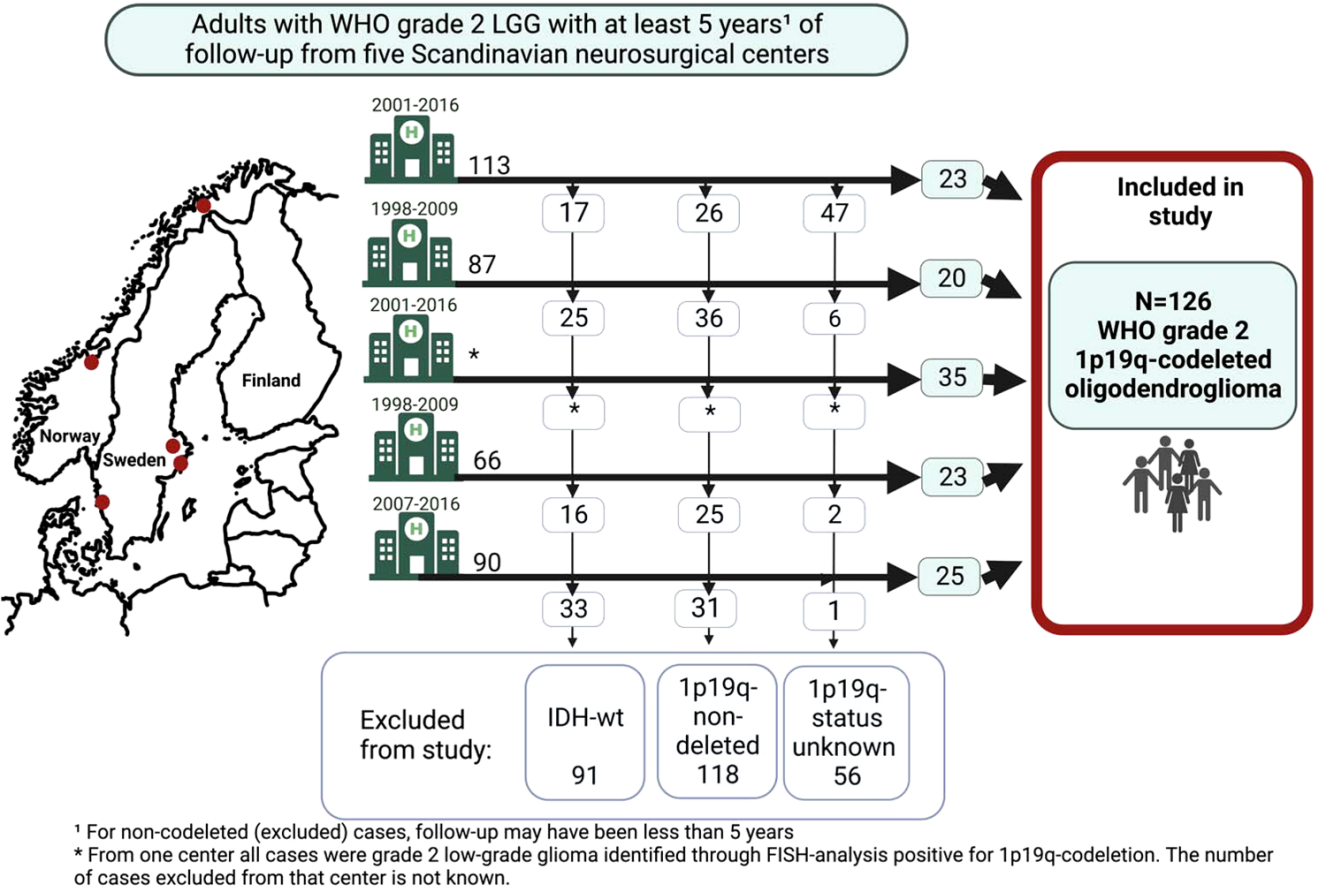
Flow chart depicting inclusion of 126 patients with 1p19q-codeleted oligodendroglioma

All adults (aged 18 or above) with a known 1p19q-codeleted oligodendroglioma WHO grade 2 diagnosis and with a minimum follow-up time of 5 years (for non-deceased patients), were included from five Scandinavian neurosurgical centers with inclusion periods between 1998 and 2016 (N = 126). Cases were retrieved from histopathological records of WHO grade 2 tumors with inclusion periods differing for the different centers but in all cases with a minimal follow-up period of 5 years and with a common end of studydate, January 1:st 2021. For details see Fig. 1. All centers serve defined geographical areas, which is why the material reflects an unselected oligodendroglioma patient population.

### Data collection

Medical records and radiological images were used to identify patient-, tumor-, and treatment characteristics. Tumor localization was defined as the cerebral lobe mainly affected. Cases with more than one lobe clearly affected were classified as multi-lobar. Eloquent tumor location was described according to Sawaya [11]. Largest diameter referred to the largest diameter in MRI measured either in the axial, coronal or sagittal plane. Regarding initial surgical strategy, patients that were initially biopsied but resected within the first 3 months after biopsy, were defined as resected. Patients never resected or resected after more than 3 months after biopsy were classified as biopsied regarding initial surgical strategy. All tumors were histopathologically identified as low grade gliomas and molecularly defined through low-grade glioma related research, or in more recent years, *IDH* and 1p19q status were detected according to clinical practice in the respective institutions. *IDH*-mutation status was evaluated with immunohistochemistry for R132H, and sequencing was used in selected cases [12]. For 1p19q detection we accepted fluorescence in situ hybridization (FISH), multiplex ligation-dependent probe amplification (MLPA) and methylation analysis as described earlier [12, 13]. All cases were 1p19q-codeleted, but 20 tumors were lacking data on *IDH* mutational status, whereas two cases were assigned to the oligodendroglioma group based upon detection of 1p19q-codeletion in the absence of detected *IDH*-mutations.

### Statistical analyses

Analyses were done with SPSS, version 28 or newer (Chicago, IL, USA) or R [version 4.2.2 GUI 1.79 High Sierra build (8160)] and R studio (version 2022.12.0 + 353). Statistical significance level was set to *p* < 0.05. All tests were two-sided. Central tendencies are presented as means ± SD, or median with first and third quartile if skewed. Overall survival and median follow-up time were estimated by the Kaplan–Meier method. Uni- and multivariable Cox regression analyses were performed for survival. Assumptions for proportional hazards were verified. For the multivariable analysis, variables were chosen by perceived clinical relevance and statistical significance in the unadjusted analysis. To avoid overloading the model, only variables associated at the *p* < 0.05 level in the unadjusted analyses were entered into the multivariable regression model. However, in a sensitivity analysis, also variables associated at the *p* < 0.1 were used for a separate multivariable model.

Kaplan–Meier curves with log rank tests were used for visualization of findings in survival analyses. Spearman´s rank correlation was used to check correlations between continuous covariates, independent t-test and Mann–Whitney U-test were used to check correlations between categorical variables and covariates when normally distributed and non-parametrically distributed respectively.

## Results

In total 126 patients were included with a median follow-up of 12.0 (CI 11.1–12.8) years (reversed Kaplan–Meier method). The median age at inclusion was 42.5 years, ranging from 20 to 78. The preoperative patient characteristics, tumor data and treatment variables are presented in Table 1.

**Table 1:** Basic clinical data in *1p19q-codeleted* WHO grade 2 oligodendroglioma patients, N = 126

**Preoperative basic variables**	
Age, median (Q1:Q3)	42.5 (34.8:53.0)
Female, n (%)	52 (41.3)
KPS preoperatively, n (%)	
100	38 (30.6)
90	54 (43.5)
80	21 (16.9)
70	11 (8.9)
Missing	2
Focal deficit preoperatively, n (%)	24 (19.0)
Seizures preoperatively, n (%)	91 (73.4)
Missing	2
**Preoperative tumor variables**	
Max tumor diameter in mm, mean (SD)	56.3 (19.2)
Missing	5
Main lobe affected n (%)	
Frontal	79 (62.7)
Temporal	11 (8.7)
Parietal	6 (4.8)
Occipital	3 (2.4)
Insular/BG	3 (2.4)
Multilobar	24 (19)
Eloquence, n (%)	
Sawaya I	50 (40.3)
Sawaya II	30 (24.2)
Sawaya III	44 (35.5)
Missing	2
Any CE, n (%)	31 (24.6)
Tumor crossing midline, n (%)	27 (21.4)
**Treatment variables**	
Initial surgical strategy, n (%)	
Biopsy	29 (23.0)
Resection	97 (77.0)
Any resection during follow-up	111 (88.1)
Time to second procedure (months) median (Q1:Q3)	51.0 (24.3:85.7)
Re-operations	
0	70 (55.6)
1	37 (29.4)
2	12 (9.5)
3 or more	7 (5.6)
Chemotherapy within 6 months n (%)	24 (19.0)
Type of chemotherapy within 6 months	
Temozolomide	10 (41.7)
PCV	4 (16.7)
CCNU	10 (41.7)
Ever chemotherapy, n (%)	80 (63.5)
Type of chemotherapy first line, n (%)	
Temozolomide	37 (29.4)
PCV	24 (19.0)
CCNU	18 (14.3)
Type of chemo missing	1 (0.8)
No chemo during follow-up	46 (36.5)
Radiotherapy within 6 months	45 (35.7)
Ever radiotherapy	90 (71.4)
**Survival**	
Deceased, n (%)	37 (29.4)
Survival years, median (95%CI)	17.8 (16.0–19.6)

KPS denotes Karnofsky Performance Status; PCV, procarbazine hydrochloride, CCNU (lomustine), and vincristine sulfate

As shown in Table 1, the cohort was heterogeneously treated; 24 patients (19.0%) had early chemotherapy, 45 patients (35.7%) had early radiotherapy, whereas 10 patients (7.9%) had both radio-and chemotherapy within 6 months. All patients but 15 (11.9%) underwent resective surgery at some point during the follow-up-period.

### Survival

**Fig. 2:**
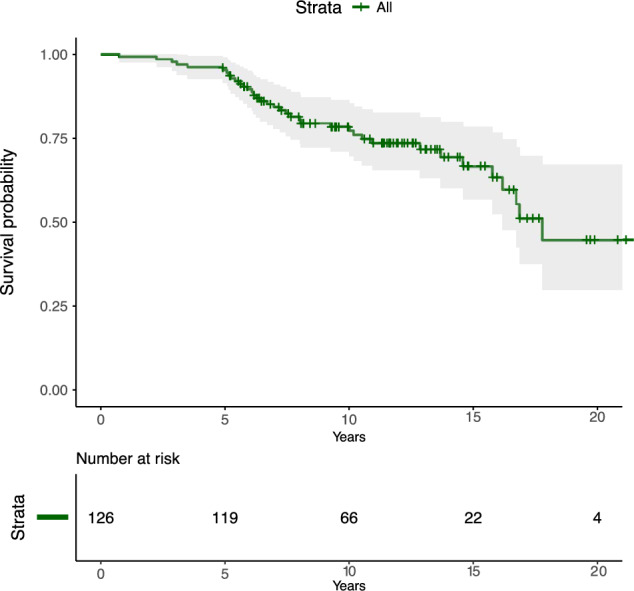
The median overall survival for all 1p19q-codeleted WHO grade 2 oligodendroglioma patients was estimated to 17.8 years (95% CI 16.0–19.6)

The median survival time was 17.8 years (Fig. 2).

### Predictors for survival

In Cox regression analysis, factors affecting survival were examined (Table 2).

**Table 2:** Predictors of impaired survival in 1p19q-codeleted WHO grade 2 oligodendrogliomas according to uni- and multivariable Cox regression analysis

Variable		Univariable analysis			Multivariable analysis		
		Unadjusted Hazard ratio	95% CI	*p*-value	Adjusted Hazard ratio	95% CI	*p*-value
Age	Per year	1.07	1.04–1.10	** < 0.00001**	1.05	1.02–1.08	** < 0.001**
Sex	Female Male	1 (ref.)					
		0.71	0.37–1.36	0.30			
KPS < 80	No Yes	1 (ref.)			1 (ref.)		
		5.27	2.32–11.94	** < 0.0001**	4.47	1.70–11.78	**0.002**
Focal deficit preoperatively	No Yes	1 (ref.)			1 (ref.)		
		2.10	1.06–4.14	**0.033**	1.42	0.62–3.27	0.41
Seizures preoperatively	No Yes	1 (ref.)					
		0.61	0.31–1.22	0.16			
Max tumor diameter	Per mm	1.05	1.03–1.07	** < 0.00001**	1.05	1.02–1.08	** < 0.001**
Eloquence according to Sawaya	I–II III	1 (ref.)					
		1.75	0.90–3.41	0.10			
Tumor crossing midline	No Yes	1 (ref.)			1 (ref.)		
		2.56	1.22–5.35	**0.013**	1.53	0.62–3.80	0.36
Contrast enhancement	No Yes	1 (ref.)					
		1.73	0.82–3.66	0.15			
Initial surgical strategy Resection biopsy	Resection Biopsy	1 (ref.)					
		1.06	0.51–2.20	0.87			
Ever resection	No Yes	1 (ref.)					
		0.99	0.39–2.55	0.99			
Chemotherapy within 6 months postop	No Yes	1 (ref.)					
		0.75	0.29–1.92	0.55			
First line chemotherapy	No chemo	1 (ref.)					
	PCV	1.14	0.42–3.10	0.80			
	Tzd	1.53	0.70–3.39	0.29			
	CCNU	1.27	0.44–3.66	0.66			
Radiotherapy within 6 months postop	No	1 (ref.)					
	Yes	1.82	0.95–3.51	0.07			

KPS denotes Karnofsky performance status; Tzd, Temozolomide; PCV, procarbazine hydrochloride; CCNU (lomustine), and vincristine sulfate

Variables associated at the *p* < 0.05 level in the univariable analyses were entered into the multivariable analysis

Bold indicate statistical significance at the *p* < 0.05-level

In univariable analyses, the parameters increased age, impaired functional status (KPS < 80), preoperative neurological deficit, tumor crossing the midline of the brain and larger tumor diameter, were correlated with reduced survival. In adjusted analysis, only increased age (HR 1.05; CI 1.02–1.08, *p* < 0.001), larger tumor diameter (HR 1.05; CI 1.02–1.08, *p* < 0.001) and KPS < 80 (HR 4.47; CI 1.70–11.78, *p* = 0.002) remained associated with shorter survival. A sensitivity analysis including all variables associated with survival at a *p* < 0.1 level did not change the results (Supplementary Table S1), nor did a corresponding multivariable model that also included "initial surgical strategy" (data not shown).

**Fig. 3:**
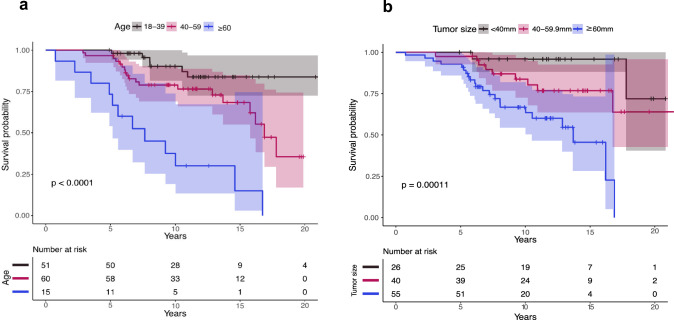
Kaplan–Meier curves illustrating impaired survival in **a** older patients (*p* = 0.0001) and **b** patients with larger maximal tumor diameter (*p* = 0.00011)

Kaplan–Meier curves are presented for age and tumor size strata to illustrate findings visually (Fig. 3).

There was no statistically significant correlation between age and tumor size (Spearman’s rho = 0.13, *p* = 0.16). Nor was the difference in median age and mean tumor size significant for patients with KPS < 80 compared to those with KPS ≥ 80 (age 51.0 vs. 42.0 years, *p* = 0.50, tumor diameter 63.6 vs. 55.8 mm, *p* = 0.20).

Sub-analyses were made assessing early chemo- and radiotherapy in groups stratified by risk (Supplementary Fig. S1). In the low-risk stratum (age < 45 and tumor size < 50 mm) there were only three events (n = 28). In the high-risk group (age ≥ 45 and tumor size ≥ 50 mm) (n = 97), there was no statistically significant difference in survival comparing early chemo- or radiotherapy with delayed or absent such therapy (Supplementary Fig. S1).

## Discussion

In this population based multi-center observational study with long-term data of WHO grade 2 oligodendrogliomas, the median survival was almost 18 years. During this long follow-up, patients were heterogeneously treated, and most patients underwent multiple treatment interventions. Only increased age, larger tumor diameter and KPS < 80 correlated with impaired survival in multivariable analyses.

### Baseline characteristics

In line with previous studies of oligodendrogliomas, the patients in the present cohort were somewhat older at diagnosis than what is typically reported for LGG cohorts that include a mixture of *IDH*-mutated astrocytomas and oligodendrogliomas. Also, more males than females were affected, the vast majority had seizures preoperatively, and the tumors had a predilection for frontal lobe engagement [10, 14–20]. We believe that this congruence with earlier observations supports the external validity of the present study.

### Prognostic factors

As seen in our results, and as previously known from earlier studies with molecularly defined oligodendrogliomas, the survival times clearly exceed those of other diffuse gliomas [10, 17, 21, 22]. Publications on oligodendrogliomas lacking molecular data have probably been subjected to considerable misclassification and therefore also to confounding effects from *IDH*-wt tumors and *IDH*-mut astrocytomas [23–25]. More recent publications with separate analyses for the different molecular tumor subtypes, on the other hand, are often disadvantaged by short follow-up times in relation to the expected survival time [10, 14, 16–18, 23, 26–35].

Bearing these limitations in mind, the present study together with several earlier studies identify older age as a predictor for poor survival also in molecularly defined cohorts [23, 28, 33, 35–37]. The correlation was however not reported in another recent large cohort study [10]. Since higher age is associated with shorter survival also among healthy individuals and the median survival in our cohort is nearly 18 years, some patients may of course have died from unrelated causes.

Studies on 1p19q-codeleted tumors presenting data on pre-operative tumor size have almost consequently shown a correlation between larger tumor size and worse prognosis [10, 14, 17, 19, 34, 36, 38]. Nevertheless, in a large registry based study by Garton et al. no significant correlation was seen between tumor size and survival [23]. This conflicting result might derive from the potentially less reliable size data in the registry of the mentioned study.

The shorter survival associated with impaired performance status (KPS < 80) seen in the current work is unsurprising and confirms earlier studies, even if it is not entirely clear whether it stands for advanced disease or serious co-morbidities that were not adjusted for [7, 28].

Initial surgical strategy (biopsy vs. resection) did not significantly affect survival in the present study. The strong correlation between Extent of resection (EOR)/Gross total resection (GTR) and survival often found in studies involving astrocytomas, seems to be less apparent (even if sometimes present) for patients with 1p19q-codeleted oligodendrogliomas [7, 10, 14, 17, 19, 26, 28, 29, 32, 39], although contrary results do exist [16].

There are also several oligodendroglioma studies with data from large American cancer registries (National Cancer Database/NCBD and SEER/Surveillance, Epidemiology and End Results) that have shown correlations between GTR and prolonged survival when compared to biopsy/no surgery [34, 35], especially in anaplastic oligodendrogliomas [23]. Conflicting results particularly for the role of subtotal resections (STR) among these studies, and in relation to other studies based on the same registries, have been reported, possibly due partly to different interpretations of the codes used for extent of resection [23, 29, 34, 35, 40, 41]. The less prominent effect of surgical resection in oligodendrogliomas may be due to data immaturity, since, in many studies, the number of events may come in single digits for the oligodendroglioma subgroup [17, 18, 27, 31] or reflect only the first few years in a disease course expected to last for almost two decades [14, 29, 33]. It could also be that these tumors are more responsive to other treatment, affecting the overall surgical impact [21]. Also, in observational data with long follow-ups, patients may undergo multiple interventions at various time points, making it difficult to isolate effects of single treatment elements. Further, in studies like ours, that lack volumetric data, a therapeutic effect of surgery may not emerge as clearly as in those with quantified residual tumor volumes, where an assumed dose response relationship would be possible to detect. In the present study, the category "resection" includes surgeries with any attempt of debulking surgery as well as complete resections due to lack of postoperative imaging in the earlier time-periods. Nevertheless, in a recent study by Hervey-Jumper et al. [10] the association between residual tumor volume and survival was clear but not independent from preoperative tumor volume in molecularly defined oligodendrogliomas (unlike the case for astrocytomas). Despite long follow-up, data immaturity was a concern also in this publication [10], making conclusions difficult to draw when only 16.3% (31/190) of the patients with oligodendrogliomas were deceased in the largest cohort with the longest follow-up.

For WHO grade 3, 1p19q-oligodendrogliomas, an interesting publication by Garnier et al. specifically addressed short-time survivors (cancer specific survival less than 5 years) [36]. These patients differed in several ways from the classical survivors and were for example older (median age 57.4), more often presented with symptoms other than only seizures, were more often biopsied, and had a larger preoperative tumor volume (mean 186 cm3). Almost all the clinical factors characterizing the short-time-survivors of this study, were also found as predictors for impaired survival in the present study, at least in univariable analysis (age, large tumor size, impaired cognition/neurological deficit, engagement of midline structures).

We believe that the long survival times demonstrated in this publication are important to consider when deciding on the best timing of treatment in relation to the risk profile [42–45].

For example, high risk resections or early radiotherapy should be weighed up against the relatively long survival on group level, thus allowing for multiple reinterventions if needed later, postponing the risk for sequelae. However, the identified risk factors for shorter survival may be useful in decision making when considering pros and cons of different treatment options.

### Limitations

The study is subject to all limitations inherent to a retrospective observational study including the inability to conclude causality from detected associations. As most patients underwent multiple interventions, the results should not be confused with the natural course of the disease. Also, the sample size of 126 patients, although one of the larger cohorts in the context of molecularly defined cases with individual level data, is limited. Similarly, and as mentioned before, the median follow-up of 12 years may, even though probably longer than in any other molecular study, still be regarded as too short, considering the expected longevity of the analyzed cohort. Finally, volumetric data on tumor residuals would have increased the resolution with which the effect of surgery could be evaluated.

## Conclusion

This long-term study of patient with heterogeneously treated 1p19q-codeleted oligodendroglioma WHO grade 2 demonstrates that the median survival approaches 20 years in a population-based setting. Further, increased patient age, lower functional status and larger preoperative tumor size were independently associated with impaired survival. Altogether, these findings may be used to weigh risks and benefits of treatment, especially considering potential long-term risks of early treatment.

## Supplementary Information

Below is the link to the electronic supplementary material.

Supplementary file 1 (DOCX 19 KB)

Supplementary file 2 (EPS 1018 KB)

## Data Availability

The data that support the findings of the present study are not publicly available, due to them containing information that could compromise research participant privacy/consent. The data are however available upon reasonable request to the authors.
