# Supplementary material for: Long-term follow up of patients with WHO grade 2 oligodendroglioma
Source: J Neurooncol. 2023 Aug 21;164(1):65–74. doi: 10.1007/s11060-023-04368-6 (PMC10462563; doi:10.1007/s11060-023-04368-6)
Supplement: Supplementary file 1 — (DOCX 19 KB) [file 11060_2023_4368_MOESM1_ESM.docx]

| Supplementary table 1. Sensitivity analysis of predictors of impaired survival in 1p19q-codeleted WHO grade 2 oligodendrogliomas. Variables associated with survival in the unadjusted analysis at the p< 0.1 level are included in the multivariable analysis. | | | | | | | |
| --- | --- | --- | --- | --- | --- | --- | --- |
| Variable | | Univariable analysis | | | Multivariable analysis | | |
|  |  | Unadjusted Hazard  ratio | 95% CI | p-value | AdjustedHazard ratio | 95% CI | p-value |
| Age | Per year | 1.07 | 1.04-1.10 | **<0.00001** | 1.05 | 1.02-1.08 | **0.001** |
| Sex | Female  Male | 1 (ref)  0.71 | 0.37-1.36 | 0.30 |  |  |  |
| KPS <80 | No  Yes | 1 (ref)  5.27 | 2.32-11.94 | **<0.0001** | 1 (ref)  4.79 | 1.65-13.90 | **0.004** |
| Focal deficit preoperatively | No  Yes | 1 (ref)  2.10 | 1.06-4.14 | **0.033** | 1 (ref)  1.36 | 0.54-3.44 | 0.51 |
| Seizures preoperatively | No  Yes | 1 (ref)  0.61 | 0.31-1.22 | 0.16 |  |  |  |
| Max tumor diameter | Per mm | 1.05 | 1.03-1.07 | **<0.00001** | 1.04 | 1.01-1.08 | **0.004** |
| Eloquence according to Sawaya | I-II  III | 1(ref)  1.75 | 0.90-3.41 | 0.10 |  |  |  |
| Tumor crossing midline | No  Yes | 1 (ref)  2.56 | 1.22-5.35 | **0.013** | 1 (ref)  1.58 | 0.59-4.20 | 0.36 |
| Contrast enhancement | No  Yes | 1 (ref)  1.73 | 0.82-3.66 | 0.15 |  |  |  |
| Initial Surgical strategy  Resection  biopsy | Resection  Biopsy | 1 (ref)  1.06 | 0.51-2.20 | 0.87 |  |  |  |
| Ever resection | No  Yes | 1 (ref)  0.99 | 0.39-2.55 | 0.99 |  |  |  |
| Chemotherapy within 6 months postop | No  Yes | 1 (ref)  0.75 | 0.29-1.92 | 0.55 |  |  |  |
| First line chemotherapy | No chemo  PCV  Tzd  CCNU | 1 (ref)  1.14  1.53  1.27 | 0.42-3.10  0.70-3.39  0.44-3.66 | 0.80  0.29  0.66 |  |  |  |
| Radiotherapy within 6 months postop | No  Yes | 1 (ref)  1.82 | 0.95-3.51 | 0.07 | 1 (ref)  1.48 | 0.65-3.34 | 0.35 |

KPS denotes Karnofsky Performance Status; CE contrast enhancement; Tzd Temozolomide; PCV procarbazine, hydrochloride, CCNU (lomustine), and vincristine sulfate

Bold figures indicate statistical significance at the p<0.05-level
